# 2182: Developing a corpus for natural language processing to identify bleeding complications among intensive care unit patients

**DOI:** 10.1017/cts.2017.60

**Published:** 2018-05-10

**Authors:** Rashmee Shah, Benjamin Steinberg, Brian Bucher, Alec Chapman, Donald Lloyd-Jones, Matthew Rondina, Wendy Chapman

**Affiliations:** The University of Utah School of Medicine, Salt Lake City, UT, USA

## Abstract

OBJECTIVES/SPECIFIC AIMS: An accurate method to identify bleeding in large populations does not exist. Our goal was to explore bleeding representation in clinical text in order to develop a natural language processing (NLP) approach to automatically identify bleeding events from clinical notes. METHODS/STUDY POPULATION: We used publicly available notes for ICU patients at high risk of bleeding (n=98,586 notes). Two physicians reviewed randomly selected notes and annotated all direct references to bleeding as “bleeding present” (BP) or “bleeding absent” (BA). Annotations were made at the mention level (if 1 specific sentence/phrase indicated BP or BA) and note level (if overall note indicated BP or BA). A third physician adjudicated discordant annotations. RESULTS/ANTICIPATED RESULTS: In 120 randomly selected notes, bleeding was mentioned 406 times with 76 distinct words. Inter-annotator agreement was 89% by the last batch of 30 notes. In total, 10 terms accounted for 65% of all bleeding mentions. We aggregated these results into 16 common stems (eg, “hemorr” for hemorrhagic and hemorrhage), which accounted for 90% of all 406 mentions. Of all 120 notes, 60% were classified as BP. The median number of stems was 5 (IQR 2, 9) in BP Versus 0 (IQR 0, 1) in BA notes. Zero bleeding mentions in a note was associated with BA (OR 28, 95% CI 6.5, 127). With 40 true negatives and 2 false negatives, the negative predictive value (NPV) of zero bleeding mentions was 95%. DISCUSSION/SIGNIFICANCE OF IMPACT: Few bleeding-related terms are used in clinical practice. Absence of these terms has a high NPV for the absence of bleeding. These results suggest that a high throughput, rules-based NLP tool to identify bleeding is feasible.

